# Brazilian solar saltworks - ancient uses and future possibilities

**DOI:** 10.1186/2046-9063-8-8

**Published:** 2012-04-10

**Authors:** Renato De Medeiros Rocha, Diógenes FS Costa, Milton A Lucena-Filho, Rodolfo M Bezerra, David HM Medeiros, Antonio M Azevedo-Silva, Cristian N Araújo, Lauro Xavier-Filho

**Affiliations:** 1 Departamento de Geografia, Universidade Federal do Rio Grande do Norte, Campus de Caicó, Joaquim Gregório, s/n, Penedo 59.300-000, Caicó-RN, Brasil; 2 Departamento de Biologia, Universidade de Aveiro, Campus de Santiago, 3810-193 Aveiro, Portugal; 3 Instituto de Tecnologia e Pesquisa, Universidade Tiradentes, Av. Murilo Dantas, 300. Bairro Farolândia, 49032-490 Aracaju, Sergipe, Brasil

**Keywords:** Wetland, Salt production, Management, Brazil, Conservation

## Abstract

Coastal solar saltworks of Brazil are exploited for sea salt, which becomes progressively concentrated by evaporation. This study aimed to review the current and new potential uses of these systems, in order to provide more dynamic for this activity. The first evaporation ponds are also used for artisanal fisheries, ensuring the livelihood of many families. All the brine rich in secondary salts (bittern) can be widely used by the chemical industry, while the Brazil shows an incipient production of "flower of salt", a salt with distinct characteristics with higher market value than sodium chloride. On the other hand, the saltponds have a high potential for management and obtaining of large populations of *Artemia *spp., purifying the brine through the action as biological filter. This microcrustacean occurs naturally in intermediate salinity ponds, being commonly used in aquaculture. Species of microalgae and halobacteria found in the saltworks are employed for extraction of beta-carotene and glycerol, used in an extensive list of products with high commercial value. These ecosystems represent refuge zones for many species of migratory birds, becoming imperative to promote the conservation of these hypersaline wetlands.

## Background

Coastal solar saltworks are anthropogenic supratidal habitats exploited for sea salt, which becomes progressively concentrated by evaporation [1]. This multi-pond ecosystem provides a range of environments with different salinity levels, from that of seawater up to sodium chloride saturation and sometimes even beyond [2]. As water evaporates and salinity increases, water is pumped or fed by gravity to the next pond, so that the salinity in each particular pond is kept within narrow limits, essentially constant [3].

Brazilian largest saltworks are located in coastlines, more specifically on the shores of estuaries in the northern coast of Rio Grande do Norte State. The establishment of the saltworks in the coastal zones of Rio Grande do Norte occurred with the use of salt plains of rivers. The saltwork ponds located in this stretch of the Brazilian coast account for 95% of sea salt produced in the country and exported, directly influencing the local and regional economies by creating jobs and payment of taxes [4].

Considering the form of exploration and harvesting of salt, we can classify the Brazilian solar saltwork ponds into two categories: craft (manual) and mechanized. The craft harvest is small, with an average area of 2-50 ha, divided into 10-20 tanks (evaporators and crystallizers), with manual harvesting of salt and a production of approximately 200-20,000 ton.year^-1^. The short retention time of the brine in each evaporator prevents the full development of a stable ecosystem [1-3,5]. The result is a salt regarded as second quality by chemical, especially its content of organic and inorganic impurities, and lead tiny crystal and brittle [5,6]. On the other hand, a typical mechanized saltwork pond usually produces over 150,000 ton.year^-1^, with a production area over 500 ha. The retention time of the brines is longer and the sea water abstracted by the saltwork arises as concentrated brine in the area of crystallization approximately three months later. In this way, this paper shows a brief review, whereby besides sodium chloride production, this system (solar saltwork) has potential for many other economic activities, being possible to associate the salt production with other multiple potential uses of the salt ponds.

## Artisanal fisheries

The consumption of fish and derivatives has been documented as having beneficial effects on human health due to the presence of omega-3 polyunsaturated fatty acid (PUFA) [7]. For the poor populations from coastal regions of the semiarid which suffer from nutritional deficiency, artisanal fisheries are one of the only sources of supply and obtaining income. In another sphere, associated with the entrance of fish in the saltworks by means of the pumping stations, the artisanal fishing in the evaporators of the saltworks is an activity that has been developed since the construction period. Several families who live near the saltworks depend directly on this activity for survival. For this, they also control the populations of fish inserted in the food chain of the saltworks ecosystems in Brazil [4].

This activity remarkably shows the direct relationship between the semi-artificial ecosystems of the saltwork [2,5] with the local fishermen. Fishing is also done in the estuary and tidal channels but, the catch of most species occurs faster and in greater quantity in the initial evaporators of the saltworks. This easiness concerns the fact that several species of fish, mollusk and crustacean are caught at juvenile stages in the pumping stations of the estuarine water, pulled into the saltworks by powerful pumps that abstract water into for these initial evaporators [1,8]. Most of these species does not survive to gradual increase of salinity along production circuit, perishing soon after the transference of water in the initial evaporators, when the salinity begins to reach 10-12° Bé. Fishermen act as important controlling elements of the ecosystem, once not caught, the dead biota are deposited in the margins of the evaporators, causing strong odor and increasing considerably the organic matter (and consequent eutrophication) inside the saltwork [4].

## Extraction of secondary salts from "Mother Waters" ("Bitterns") used for chemical industry

The sequence of salts deposited by the evaporation of sea water is in accordance to the solubility of its several compounds. Thus, the precipitation of salts includes the less soluble compounds in the base to the more soluble at the top of the sequence, in the following order: limestone (CaCO_3_), gypsum (CaSO_4_), halite (NaCl), potassium salts sylvinite (NaCl-KCl system), and magnesium salts (bischofite - MgCl_2_·6H_2_O); it is also considered the presence of other compounds, according to physical and chemical variations of the brine during the various stages of evaporation [9].

NaCl crystal (halite) is formed when the total salt concentration reaches value above 300 gL^-1^. After most of the NaCl precipitates to the bottom of the crystallizer ponds, the remaining concentrated brine (the "bitterns") contains mainly Mg^2+^, K^+^, Cl^- ^and SO_4_^2- ^(Oren, 2002). The bittern remaining after the crystallization of halite is nutrient-rich, but apparently devoid of life, as no organisms tolerate the extremely high Mg^2+ ^concentration [10].

When all calcium carbonate, calcium sulfate, and 83% of the halite is crystallized from seawater by solar concentration, a bittern of a specific gravity of about 1.26 is obtained. This bittern with few exceptions is placed back to the sea. In some cases, as in Spain, is used for recovering some epsomite, bischofite, and bromine, but not for the production of potassium salts. On further evaporation, a complex mixture of halite, sylvite, and double salts of potassium, sodium, and magnesium start to crystallize; the recovery of marketable products becomes difficult and inefficient. However, in the absence or near absence of sulfate, the bittern may be readily processed to recover high-purity sylvite and bischofite with excellent efficiency [11].

The partially desulfated seawater bittern obtained from the epsomite plant is readily amenable for recovering sylvite and magnesium chloride hexahydrate by a combination of solar evaporation and fractional crystallization. Despite the very complex chemical phase of seawater bittern, a simple crystallization method may be employed for the efficient recovery of high-purity epsomite and sylvite [11].

Magnesium is profusely present in seawater evaporites as chloride (9.44%), sulfate (6.5%) and bromide (0.22%). The raw material of the magnesium industry is, however, magnesium hydroxide. This is then treated with hydrochloric acid to obtain magnesium chloride. The potential value of magnesium chloride as raw material is established, but involves separation of different salts to obtain magnesium chloride in a relatively pure form. Magnesium chloride occurs in the nature as bischofite (MgCl2 • 61120) and as carnallite (KCI • MgC12 • 6H20), both from oceanic origin [12].

The production of crystalline magnesium chloride hexahydrate by solar evaporation of low-sulfate-containing inland bittern has yielded a product suitable for electrolytic production of magnesium metal. Using the sea bittern for the production of such crystalline magnesium chloride hexahydrate was not attempted, probably due to the high value of sulfate content of about 3.5 per cent at sp. gr. 1.350. In the arid and semiarid tropical regions, solar evaporation of sea bittern reaches the equilibrium density of sp. gr. 1.377, and at equilibrium relative humidity of 32% [13]. This clearly approaches the equilibrium of the pure system of magnesium chloride hexahydrate and water enabling to take advantage of solar evaporation in the process [12].

## "Flower of salt" production

"Flower of salt" is a thin layer that forms on the surface of the salt tide, during the continuous evaporation. The salt does not suffer any transformation, besides the natural drying in the sun, which eliminates the rose tone. The flower of salt contains all 84 trace elements and micronutrients found in the sea, being a natural source of potassium, calcium, copper, zinc and magnesium [14]. An adequate level of this salt is very important for the body functioning, and is highly requested by the market of international gastronomy, replacing the refined salt [15].

This mineral product has extremely white color, with rigid crystalline structure and high moisture content. Although apparently made up by small crystals of salt, actually this form of halite has a structure organized into microcrystal clusters.

In relation to the production, this mineral is formed on the brine surface, only in a thin layer of salt crystals, which are harvested daily and dried in the sun. The harvesting is made daily on the hottest days in traditional saltworks [14]. The flower of salt is packed with no other processing, unlike what happens to with the sea salt for consumption that undergoes a process of washing, centrifugation and drying by the heat of combustion, ground and sieved.

The importance given to this product concerns the area required for production. While large saltwork companies need several kilometers for installations, operated by business groups, the flower of salt can be obtained in ponds with total area smaller than 01 hectare. Another fact concerns the production: since it is a handmade product, these small salterns can be operated by familiar groups, becoming a new income source for populations living in hypersaline areas of the country, or even to innovate small artisanal salterns still remaining.

## Mass culture of artemia for aquaculture

With the development of fish and shellfish hatchery aquaculture, the use of the brine shrimp *Artemia *as a diet for larval culture of many species has become widespread due to convenience of use and high nutritional value [16]. Dormant cysts of *Artemia *are available year-round in large quantities along the shorelines of hypersaline lakes, coastal lagoons and solar saltworks spread over the five continents [7].

After harvesting and processing, cysts are made available in cans and stored 'on demand' live food. However, the expansion of aquaculture production made the demand for *Artemia *cysts now exceeds the supply. Prices have risen exponentially, turning *Artemia *into a bottleneck for the expansion of the hatchery aquaculture of marine fish and crustacean. In particular, many developing countries can barely afford to import the very expensive cysts [7].

The use of a device such a Solar Pond (a green and renewable energy source) would save energy and time, in speeding up both *Artemia *cysts hatching time and *Artemia *nauplii development. Since the newly hatched *Artemia *nauplii are attracted to light, they easily concentrate in one area allowing the harvesting by shining a flashlight at the exit of the bioreactor.

## Microalgae and halobacteria cultures for extraction of beta-carotenes and glycerol

The halophilic unicellular green algae *Dunaliella *is grown worldwide as a source of valuable chemicals. The most important product is β-carotene but other uses have been explored as well, including the production of glycerol and the pyrolysis of *Dunaliella *biomass for the production of oil [17].

The positive effect of the presence of dense communities of red halophilic Archaea in saltern crystallizer ponds has been recognized for a long time. The red coloration that develops in these ponds is mainly caused by Archaea, but strains of *Dunaliella*, and possibly even red halophilic bacteria of the genus *Salinibacter *contribute as well toward the absorption of light energy. By trapping solar radiation these microorganisms raise the temperature of the brine and the rate of evaporation, thereby increasing salt production [2,10].

In order to improve salt production in salterns without enough dense archaeal community, the fertilization with organic nutrient has been suggested [2]. C50 bacterioruberin derivatives are the main carotenoids of the Halobacteriaceae. However, additional carotenoids may be present that have proven economic value. An isolate from a seawater evaporation pond near Alexandria, Egypt, produces considerable amounts of the *ketocarotenoid canthaxanthin *[18]. Exploitation of this organism for commercial canthaxanthin production has already been suggested [18].

*Dunaliella *protein has a similar composition to soybean meal, but with higher lysine content [19]. It is therefore suitable for use as feedstock in mariculture (crab, shrimp, shellfish) and for livestock such as chickens. As a result of the absence of cell walls, the cells are digestible [17]. Catalytic pyrolysis of *Dunaliella *cell material at 200-240°C produces an oil-like substance. The overall process is exothermic, and thus most of the thermal energy required to initiate the reaction may be regained. A conversion of 22.3% of the algal protein was obtained at 350°C to a product that contains 69.9% carbon, 7.7% hydrogen, and 7.3% nitrogen. Addition of KCl, MgCl_2 _and MnCl_2 _increased the yield to 27% with 75.5% carbon, 8.5% hydrogen, and 6.8% nitrogen [20]. At an estimated price of about $ 40 per barrel [21] this process is not economically feasible at present.

*Dunaliella *is also being used as an additive in cosmetic anti-wrinkle skin creams in combination with Dead Sea minerals [22]. The algal cell preparation allegedly binds Ca^2+ ^and Mg^2+ ^ions. However, the authors stated that "the low biosorption of calcium and magnesium obtained from the algal biomass, and the tendency to a low release of minerals at the normal pH of human skin (5.5) led to the conclusion that the advantage of these algae as a mineral vehicle for Ca and Mg is limited [17]."

## The role of biological system on maintenance of brine quality

The study on phototrophic communities inhabiting salterns is not only of purely scientific interest: the benthic cyanobacterial mats that develop in saltern ponds of intermediate salinity effectively seal the bottom of these ponds and prevent leakage of brine; on the other hand, unicellular Cyanobacteria in these mats and in the brine sometimes produce massive amounts of polysaccharide slime that unfavorably affects the salt production process [5,23].

The red pigmentation of the dense microbial communities in crystallizer ponds is caused by both the β-carotene accumulated by the green algae *Dunaliella salina*, which is the main or sole primary producer in these ponds, as well as by the carotenoid and retinal protein-based pigments of the heterotrophic community of prokaryotes that develop at the expense of photosynthetically fixed carbon derived from *Dunaliella *[24]. This red pigmentation increases light absorption by the brine and increases its temperature, thus enhancing the salt production process [10]. Even purely aesthetic considerations have been used as incentive to study the highly diverse communities of phototrophic microorganisms in salterns [5].

Recently assertions were made that the halophilic Archaea present in the crystallizer brines may be directly involved in the formation of halite crystals. It was suggested that halobacteria influence crystal growth rate and crystal habit, and that the cells and their envelope S-layers may serve as templates in the nucleation and halite crystal formation [25].

The indispensability of *Artemia *(brine shrimp) for the salt production lies on the ability of the animals to clear brine from particles up to 50 micrometers diameter, to metabolize large amounts of ingested organic matter to carbon dioxide, to deposit wastes in fecal pellets that become incorporated in the benthic community, and to provide highly suitable food for the *Halobacterium salinarium *populations in the downstream ponds [6].

Extracellular polysaccharide production by the cyanobacteria may be activated as a result of nutrient limitation as a way to dispose of the excessive fixed carbon photosynthetically produced [26]. A prominent feature of the microbial mats within the gypsum crusts, as well as in the evaporation ponds of lower salinity, is the often copious amounts of polysaccharide slime associated with the growth of the unicellular Euhalothece-Aphanothece Cyanobacteria. These organisms also spread into the overlying water in some saltern systems [5]. Massive slime formation can negatively affect the salt production process [6,26]. To control excessive blooms of these cyanobacteria, the introduction of grazing brine shrimp (*Artemia*) has been suggested as an effective management procedure [5,6].

## Solar saltworks as refuge zones for migratory birds

In a scenario of intensive occupation of coastal zone, these large aquatic ecosystems represent important refuge zones for many species of migratory birds beyond being habitat for many endemic species of hypersaline environments.

Throughout the world, certain waterbirds use saltworks as places for rest, feeding and breeding [1,27]. This guild of species is one of the most frequently considered with regard to appraisal the natural value of these wetlands for conservation as protected areas.

Salt work ponds are considered to be high-quality feeding habitats for many non-breeding shorebird species, merely based on the high number of feeding birds that they support, but it is possible that birds could also be found at high densities in habitats of low quality. There are empirical confirmation that saltworks are indeed suitable feeding habitats for several migrating shorebird species that rely on intertidal habitats [27].

When saltworks are found near important wintering and/or staging areas, further saltworks loss could cause a movement and even an increase in the mortality of the displaced birds through density dependent forces. At Cadiz Bay, for example, the coincidence of saltworks loss, and the decline and redistribution of some shorebird species has led to the suggestion of a causal link between them [28].

From a functional point of view, the key factor for the Mediterranean saltworks is the gradient of salinity. The salt production process determines ecological partitioning within the system. This ecological segregation is very important for conservation of these environments because spatial heterogeneity can provide to species a high diversity of habitats, suitable for migratory waterbirds. Such habitats are nodes of ecological connectivity [1].

## Conclusion

There is an urgent need to establish a strategy that promotes the inclusion of the Brazilian solar saltworks as conservation zones, in whose boundaries only can be developed activities that do not damage the ecological stability of these important and unique ecosystems. These environments have an ecological dynamics in space and time, where the existing knowledge about the diversity and potential use of natural resources found are still incipient. Therefore, the integrated management of solar saltwork ponds has implied the need for ongoing monitoring and conducting further studies on the feasibility of these other potential uses in Brazilian saltwork ponds.

## Competing interests

The authors declare that they have no competing interests.

## Authors' contributions

Considering the continental area of Brazil, this analyze needed of a research group of diverse areas. All review it was write by RMR, LXF and DFSC, but they work specifically in themes about "Microalgae and halobacteria cultures for extraction of beta-carotenes and glycerol", "Mass culture of Artemia for aquaculture" and "The role of biological system on maintenance of brine quality". For other side, all the authors help us during bibliographical research. RMB are responsible for the topic of "Artisanal fisheries", DHMM for "Extraction of secondary salts from ≈Mother Waters≈ (Bitterns) used for chemical industry", AMAS for the theme "Flower of salt production", and MALF and CNA coleted many data and references about the "Solar saltworks as refuge zones for migratory birds". "All authors read and approved the final manuscript".
